# Evaluation of tissue-engineered blood vessels as three-dimensional in vitro testing system in cardiovascular research

**DOI:** 10.3389/fbioe.2026.1729469

**Published:** 2026-05-18

**Authors:** Diana M. Rojas-González, Frederic Wolf, Nicole Schaaps, Roberta A. Florescu, Carolina Bienzeisler, Rahma Shahin, Celine Brinkmann, Pakhwan Nilcham, Felix Vogt, Stefan Jockenhoevel, Anne Turoni-Glitz, Petra Mela

**Affiliations:** 1 Department of Biohybrid and Medical Textiles (BioTex) at Center of Biohybrid Medical Systems (CBMS), AME-Institute of Applied Medical Engineering, Helmholtz Institute, RWTH Aachen University, Aachen, Germany; 2 Technical University of Munich, School of Engineering and Design, Department of Mechanical Engineering, Medical Materials and Implants, Munich Institute for Biomedical Engineering, Garching, Germany; 3 Department of Cardiology, Intensive Care, and Vascular Medicine, University Hospital RWTH Aachen, Aachen, Germany

**Keywords:** cellular crosstalk, *in vitro* testing, intimal hyperplasia, stent implantation, tissue engineering

## Abstract

**Background:**

Disturbed crosstalk between endothelial cells (ECs) and vascular smooth muscle cells (SMCs) has an important role in intimal hyperplasia and restenosis after vascular interventions, however, the exact pathomechanisms are incompletely understood. Current preclinical models inadequately recapitulate the complexity of human arteries.

**Objective:**

We present tissue-engineered blood vessels (TEBVs) as a novel *in vitro* model and validate it for intimal hyperplasia.

**Methods:**

TEBVs fabricated from SMC suspended in fibrin gel, supported by a textile mesh, were seeded with ECs at various concentrations and subjected to arterial flow conditions in a bioreactor for 21 days. In addition, TEBVs underwent plain old balloon angioplasty (POBA) and implantation of bare metal stents (BMS) and drug-eluting stents (DES) at day 7 after fabrication. TEBVs were monitored by optical coherence tomography.

**Results:**

TEBVs with absent or incomplete endothelial layer exhibited thicker vessel walls, more disorganized collagen. Quantitative analysis of Ki67 and αSMA revealed no statistically significant differences in SMC proliferation or contractile phenotype across varying degrees of endothelial coverage. POBA and stent implantation were feasible at day 7. 14 days post-intervention, POBA-treated TEBVs exhibited significantly thicker vessel walls than untreated controls and stented TEBVs, whereas stented TEBVs showed greater lumen diameters than unstented TEBVs. Endothelial coverage was higher in BMS than DES. Levels of IL-6, IL-8, and MCP-1 were highest in medium from BMS-treated TEBVs.

**Conclusion:**

TEBVs provide a promising *in vitro* platform to study intimal hyperplasia. Their dimensions and wall thickness resemble human coronary arteries, making them suitable for testing new stent designs.

## Graphical Abstract

**Figure:**
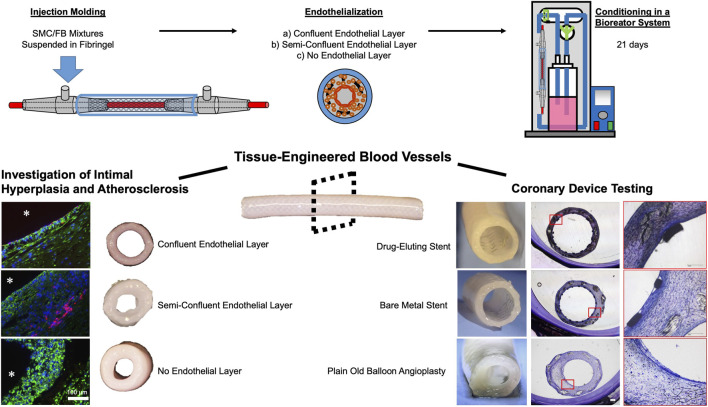
Tissue-engineered blood vessels (TEBV) fabricated from smooth muscle cell/fibroblast mixtures suspended in fibrin gel, supported by a textile mesh, were seeded with endothelial cells and conditioned in a bioreactor system for 21 days. Different endothelialization strategies resulted in differences in wall thickness. In addition, TEBVs underwent plain old balloon angioplasty (POBA) and stent implantation. POBA-treated TEBVs exhibited thicker vessel walls compared with non-treated TEBV controls and compared with stented TEBVs. We also observed significantly higher stent strut coverage with endothelial cells after implantation of bare metal stents (BMS) compared to drug-eluting stents (DES). TEBVs are a promising approach towards an *in-vitro* system for the study of intimal hyperplasia. Due to their similarity in size and wall thickness to human coronary arteries, TEBVs may also serve as a platform for testing new stent designs.

## 1 Introduction

Arterial vessel walls are continuously exposed to mechanical, hemodynamic, and neurohumoral stimuli. Orchestrated by the interplay between cells within the arterial wall, both physiologic and pathologic stimuli elicit adaptive changes in structure and functionality of the vascular wall.

The integrity of the innermost layer of the vessel wall, the vascular endothelium, is a prerequisite for vascular homeostasis. Vascular endothelial cells (EC) act as a barrier between vessel wall and blood stream, regulate the passage of macromolecules, and control the vascular tone. The endothelium coordinates inflammation by mediating the recruitment of inflammatory cells and platelets and keeps the balance between coagulation and fibrinolysis. Disturbances of normal endothelial functions, however, may pave the way for the initiation and progression of intimal hyperplasia (IH) and atherosclerosis (Ross, 1999; Brunner et al., 2005).

Vascular smooth muscle cells (SMCs) in the vascular media exhibit remarkable plasticity. While SMCs have a contractile phenotype under physiological conditions, they can differentiate to a synthetic state, along with increased abilities to migrate and to proliferate in response to injury (Shi et al., 2019). These phenotypic and functional transitions in vascular SMCs have a major role in arterial remodeling (Shi et al., 2019).

The crosstalk between ECs and SMCs is crucial for vascular homeostasis and has critical impact in vascular disease (Lilly, 2014; Li et al., 2018; Méndez-Barbero et al., 2021). ECs and SMCs interact directly via cell-to-cell contacts and indirectly via extracellular matrix (ECM) proteins or through secreted molecules and extracellular vesicles (Lilly, 2014; Sandoo et al., 2010). Importantly, a continuous release of vasoactive compounds by ECs is required to maintain SMCs in a contractile and quiescent state (Li et al., 2018; Fillinger et al., 1997; Powell et al., 1998; Korff et al., 2001). Endothelial dysfunction or loss from mechanical or chemical injury, a key event in atherogenesis (Ross, 1999), disrupts EC-SMC interactions. Furthermore, catheter-based interventional procedures such as plain old balloon angioplasty (POBA) and stent implantation inevitably lead to endothelial denudation (Pasternak et al., 1980; Block et al., 1981; Cornelissen and Vogt, 2019). The consequential loss of a continuous and functional endothelial layer may result in SMC proliferation and their transition into a synthetic phenotype with subsequent accumulation of ECM proteins, mainly collagen-I and collagen III, within the vascular wall (Méndez-Barbero et al., 2021; Libby, 2009; Xu and Shi, 2014). Therefore, disturbed crosstalk between ECs and SMCs is an important etiologic factor in IH, and in the development of restenosis after POBA and stent implantation (Nagler et al., 1997).

However, the exact pathomechanisms of how disturbed EC-SMC communication may trigger atherogenesis and restenosis are incompletely understood. While abundant studies have investigated the functions of ECs and SMCs as independent entities, a more accurate *in vitro* analysis of the pathophysiology of blood vessels requires co-culture systems of ECs and SMCs. A variety of co-culture setups have been introduced over time (Fillinger et al., 1997; Powell et al., 1998; Korff et al., 2001; van Buul-Wortelboer et al., 1986; Graham et al., 1991; Fillinger et al., 1993; Wallace et al., 2007; Ganesan et al., 2017; Chiu et al., 2004).

Two-dimensional bilayer models of ECs and SMCs in co-culture are relatively cheap and allow for very basic investigation of EC-SMC interactions. While bilayer models are important for initial drug- and permeability testing, they are not ideal to gain full insight into cellular phenotypes and behavior under physiologic and pathologic flow and culture conditions (Wolf et al., 2016; Barron et al., 2007; Fitzgerald et al., 2015; Ryan et al., 2016; Henderson et al., 2020). Preclinical animal models on the other hand are still a prerequisite for the regulatory approval of new devices and pharmaceutics. However, according to the principle of the 3R’s (replacement, reduction, and refinement), the use of animals for medical research gets more and more restricted (Ryan et al., 2016). Moreover, animal research is associated with high costs. An improved *in vitro* testing environment may help reduce the number of animals used in research. Moreover, a three-dimensional tubular construct of human coronary artery-like dimensions, composed of ECs and SMCs in co-culture and amenable to vascular intervention, could enable the investigation of EC-SMC-interactions following POBA and stent implantation, thereby providing a valuable platform for preclinical testing of coronary devices such as coronary stents.

Here, we present tissue-engineered blood vessels (TEBV) fabricated from SMC/fibroblast (FB) mixtures suspended in a matrix of fibrin gel and supported by a textile mesh. TEBVs were seeded with ECs at different concentrations to investigate EC-SMC interactions and IH development and subjected to arterial flow conditions in a bioreactor. In addition, TEBVs with a confluent endothelial layer underwent POBA-treatment and implantation of bare metal stents (BMS) and drug-eluting stents (DES) to evaluate their suitability as preclinical model for coronary interventions. TEBVs were monitored using optical coherence tomography (OCT).

## 2 Materials and methods

### 2.1 Cell isolation and culture

ECs and a mixed population of SMCs and FBs were isolated from human umbilical cord arteries provided by the Department of Gynecology at University Hospital Aachen, with informed consent and approval by the Ethics Committee at the RWTH Aachen Faculty of Medicine (EK 2067).

ECs were harvested by collagenase digestion (1 mg/mL collagenase, Sigma-Aldrich, Cat.# SCR103) and cultured on 2% gelatin-coated flasks in EC growth medium (EBM-2, Cat.# CC-3156, supplemented with EGM-2 SingleQuots, Cat.# CC-4176, Lonza). SMCs and FBs were obtained from 1 mm arterial rings, cultured in Dulbecco’s Modified Eagle Medium (DMEM; Gibco, Cat.# 11965092) with 10% fetal calf serum (FCS; Thermo Fisher Scientific, Cat.# A5209502) and subcultured at 80%–90% confluence. Cells from passages 2–3 (ECs) and 5–7 (SMCs/FBs) were used.

### 2.2 Fibrin gel

Lyophilized fibrinogen (Calbiochem, Cat.# 341576) was dissolved in Milli-Q water (Millipore), dialyzed overnight against tris-buffered saline (TBS; pH 7.4) using a membrane with a molecular weight cut-off of 6,000–8,000 kDa (Novodirect, Cat.# 0289062), and filter-sterilized. Fibrinogen concentration was determined by absorbance measurement at 280 nm using an Infinite M200 spectrophotometer (Tecan Group Ltd) and adjusted to 10 mg/mL.

SMC/FB mixtures were suspended in 1.4 mL TBS at a concentration of 10 × 10^6^ cells/mL. To fabricate one TEBV of 5 cm in length, 4 mL of fibrin gel was prepared from 2 mL fibrinogen solution, 1.4 mL SMC/FB mixtures in TBS, 0.3 mL 50 mM CaCl_2_ (Sigma) in TBS, and 0.3 mL 40 U/mL thrombin (Sigma).

### 2.3 TEBV fabrication and conditioning

**FIGURE 1:**
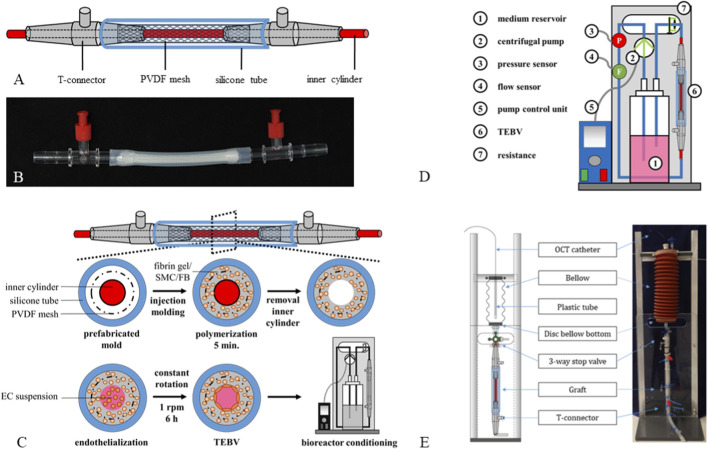
Experimental Setup. **(A,B)** Graphic illustrations and photographs of the molding system. **(C)** TEBV fabrication. SMCs/FBs in a fibrinogen-CaCl_2_-thrombin mixture were cast into the mold for gel polymerization. After removal of the inner cylinder the lumen was filled with ECs suspended in culture medium, and TEBVs were constantly rotated at 1 rpm for 6 h to uniformly distribute ECs on the inner lumen surface. TEBVs were transferred into the VascuTrainer bioreactor system for conditioning. **(D)** Graphic illustration of the VascuTrainer bioreactor system which was assembled from a medium reservoir and silicone tubing and connected to a small centrifugal pump. **(E)** A sterile module was implemented to allow for sterile introduction of balloon catheters, stents and catheters for OCT.

TEBVs were prepared using a mold as described previously (Wolf et al., 2018; Mohapatra et al., 2022), consisting of a 6.4 mm inner diameter silicone tube (Carl Roth, Cat.# CH28.1) with T-connectors (Fleima Plastic, Wald-Michelbach, Germany) at both ends and a 3 mm stainless-steel inner cylinder (Figures 1A,B). For mechanical support, a polyvinylidene difluoride mesh was fixed between the T-connectors. Fibrin gel components were inserted into the annular space to initiate polymerization. Subsequently, the inner cylinder was removed, and the lumen was seeded with ECs. TEBVs were rotated at 1 rpm for 6 h using a modified peristaltic pump (Ismatec, Wertheim, Germany) to distribute ECs evenly, then connected to the VascuTrainer bioreactor for conditioning, as described previously (Wolf et al., 2018) (Figure 1C). Briefly, the VascuTrainer consisted of a medium reservoir (Thermo Scientific) and silicone tubing (Ismatec) connected to a small centrifugal pump (702–6,882, RS components), which was controlled by a custom-developed control-unit (Figure 1D). All TEBVs were maintained at 37 °C and 5% CO_2_.

To allow for sterile introduction of balloon catheters, stents, and catheters for OCT, a sterile module, that was accessed through a disinfectable three-way valve, was implemented (Figure 1E). The module was connected to the TEBV-containing mold via a three-way stopcock. Two metal discs were used to seal the cavity at both ends and to maintain sterility. A central hole with a diameter of 2 mm within these discs enabled the introduction and centering of various catheters.

Bioreactor medium was a 50:50 mixture of DMEM and EGM-2 with 10% FCS, 1% antibiotic-antimycotic solution (ABM, Gibco, Cat.# 15240096), 1.6 μL/mL tranexamic acid (Cyklokapron® injection solution 1,000 mg/mL; Pfizer Pharma GmbH, Cat.# NDC 0013-1114-21), and 1.0 mM L-ascorbic acid-2-phosphate (Sigma; Cat.# 2004011). TEBVs were conditioned for 14 days under pulsatile flow (70 bpm) at arterial shear stress (10 dynes/cm^2^) and pressure (80–120 mmHg).

### 2.4 Hemodynamic characterization

Intraluminal wall shear stress was calculated using the Hagen-Poiseuille equation (Equation 1). This equation applies for laminar flow through a cylinder.

$$\tau =\frac{4\mu Q}{\mu r^{3}}$$ (1)

Here, µ is the dynamic viscosity, Q the volumetric flow rate, and r the radius of the cylinder, in our case, the vascular grafts. A dynamic viscosity of 0.8 mPa*s for culture media at 37 °C was used (Poon, 2022). According to Equation 1, for a TEBV with a 3 mm inner diameter and with a flow of 200 mL/min, a shear stress of 10 dyne/cm^2^ was induced.

In addition, the flow regime was characterized using the Reynolds number (Re) (Equation 2).

$$Re=\frac{4\rho Q}{\pi \mu D}$$ (2)

Here, ρ refers to medium density, which was assumed to be 1,000 kg/m^3^ (Poon, 2022). The calculated Re for a TEBV after fabrication (3 mm inner diameter), was 1768. For flow in straight circular tubes, a Re < 2000 corresponds to a laminar flow (White, 2006), which is here the case. As changes in intravascular diameter could have induced a turbulent flow, we calculated the Re for the different tested conditions using the mean luminal diameter measured at the end of the conditioning/treatment period, using an average flow of 200 mL/min. Both flow and pressure were continuously monitored during the duration of the experiments.

### 2.5 Endothelialization conditions

**FIGURE 2:**
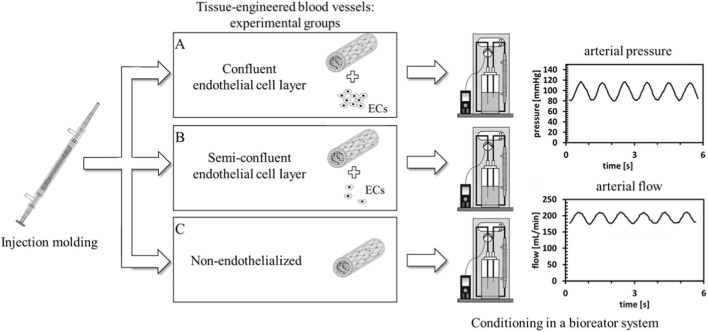
Experimental Conditions. To explore the impact of the endothelial layer on intimal hyperplasia, we compared three different endothelialization conditions of TEBVs. **(A)** ECs were seeded at a concentration of 3 × 10^6^ cells/mL to generate a confluent EC luminal layer. **(B)** To simulate a semi-confluent endothelium, TEBV were fabricated with ECs seeded at a concentration of 0.5 × 10^6^ cells/mL. **(C)** TEBV were conditioned without an EC layer. All vessels were subjected to the same arterial pressure and flow conditions.

To assess the effect of endothelialization on IH, three conditions were tested (Figure 2): (A) TEBV with a confluent EC layer (3 × 10^6^ cells/mL), (B) TEBV with a semi-confluent EC layer (0.5 × 10^6^ cells/mL), and (C) no ECs. Experiments were done in triplicate. EC confluence was confirmed via CD31 staining.

### 2.6 Balloon angioplasty and stent implantation

POBA and stent implantation were performed in TEBVs with confluent EC layers 7 days after fabrication. For POBA, 3.5 mm TREK balloons (Abbott, Cat.# 1012451) were used. Stent implantation was performed using balloon-mounted BMS (Coroflex Blue Neo, B. Braun, Cat.# 5029084) and DES (Promus Element Plus, Boston Scientific, Cat.# H7493911428350). All experiments were performed in triplicate.

Using a guidewire (HI-TORQUE Balance Middle Weight Universal Guide Wire, Abbott, Cat.# 1009662J), a balloon or balloon-mounted coronary stent was introduced into the TEBV lumen and placed 1 cm below the connector end used as point of access. Balloon inflation and stent deployment were performed at nominal pressures (Supplementary Table S1), using an insufflator (Encore™, Boston Scientific, Cat.# M0067101140). Untreated TEBVs with confluent EC layer served as control. Following POBA or stent implantation, TEBVs were conditioned for additional 14 days (*i.e.*, 21 days in total).

### 2.7 Imaging

Non-invasive external ultrasound monitoring of TEBV integrity and visualization of stent position over the course of the conditioning period was performed using a portable ultrasound system, Vivid-I (S/N 3642VI, General Electric), equipped with a linear probe (8L-RS, General Electric).

Intravascular imaging with OCT was performed after 14 days of conditioning using the Dragonfly OpStar™ Imaging Catheter with the OPTIS™ Imaging System (both Abbott).

Macroscopic images of the cross-section of the TEBVs with different degrees of endothelialization, were captured using a digital camera (Nikon Corporation, Japan). Each image was analyzed using Fiji (ImageJ) (Schindelin et al., 2012), and wall thickness was calculated as the mean of 12 measurements taken at different positions around the vessel circumference for each sample.

### 2.8 Histological and immunohistochemical analysis

TEBVs were immersion-fixed in Carnoy’s solution (VWR, Cat.# 470300–670), dehydrated, paraffin-embedded, and sectioned. Sections were deparaffinized, antigen retrieved by steam heat in citrate buffer (pH 6.0), treated with 3% H_2_O_2_. Non-specific antibody binding sites were blocked using Dako protein block (Dako, Cat.# X0909) before incubation with anti-CD31 (mouse monoclonal anti-CD31 (1:40; Dako, Cat.# M0823)). Antibody binding was detected using streptavidin-HRP (Dako, Cat.# P039701-2).

Stented and POBA-treated TEBVs, as well as their untreated control TEBVs, were fixed with 4% formalin (Carl Roth, Cat.# 4979.3) and embedded in polymethyl-methacrylate (PMMA; Technovit 9100, Kulzer, Cat.# 12225.F1000). Sequential sections from the proximal, mid, and distal parts of the stented TEBVs were sawed and grinded to sections of 40–50 µm thickness using EXACT thin section cutting system (EXACT). Sections of all TEBVs were stained with toluidine blue/basic fuchsine solution, according to the manufacturer’s instructions.

Images were obtained from cross-sections using a DMI-3000 microscope (Leica) and DISKUS image analysis software (version 4.80, C. Hilgers Technical Bureau). Measurement of lumen and wall thickness was performed by tracing the contours of the outer TEBV wall and of the lumen area using a Tavla pen tablet (Braun Photo Technik). In stented TEBVs, neointimal growth was measured above the stent struts.

### 2.9 Immunofluorescence staining

Paraffin-embedded TEBVs were deparaffinized, non-specific binding sites were blocked and the cells were permeabilized by incubation in 5% normal goat serum (NGS, Dako, Cat.# X0907) in 0.1% Triton-X-100 (Sigma, Cat.# X100) in PBS. Sections were incubated for 1h at 37 °C with the following primary antibodies: mouse monoclonal anti-α-SMA (1:1,000; Sigma, Cat.# A2547), rabbit anti-type-I collagen (1:100; Acris Antibodies GmbH, Cat.# AF5610-1), mouse monoclonal anti-CD31 (1:100; Sigma, Cat.# P8590), mouse monoclonal anti-ICAM-1 (Abcam, Cat.# AB2213), rabbit monoclonal anti-VCAM-1 (Abcam, Cat.# AB134047), rabbit polyclonal anti-eNOS (Invitrogen, Cat.# PA3-031A), rabbit polyclonal anti-iNOS (Invitrogen, Cat.# PA1-036), mouse monoclonal anti-vimentin (Dako, Cat.# M0725), mouse monoclonal anti-CD45 (Dako, Cat.# M0701), mouse monoclonal anti-CD68 (Dako, Cat.# M0814), and rabbit monoclonal anti-Ki67 (1:100; Abcam, Cat.# Ab15580). The sections were then incubated for 1 h at room temperature with either rhodamine- (Cat.# A11032) or fluorescein- (Cat.# F2765) conjugated secondary antibodies (1:400; Molecular Probes). Cell nuclei were counterstained using DAPI nucleic acid stain (Molecular Probes, Cat.# 62248). As negative controls, samples were incubated in diluent and secondary antibody only, and samples from umbilical cord artery served as positive controls. Images were acquired with a fluorescence microscope (AxioObserver Z1; Carl Zeiss GmbH, Oberkochen, Germany), equipped with a digital color camera (AxioCam MRm; Carl Zeiss GmbH).

### 2.10 Collagen content quantification

Collagen levels were determined as described before (Reddy and Enwemeka, 1996) by measuring the hydroxyproline content of overnight vacuum-dried samples from each TEBV. Briefly, samples were hydrolyzed in 500 µL of 6M hydrochloric acid (HCL) at 110 °C for 18 h. Buffered chloramine-T reagent (60 mM, 20% n-propanol and 80% acetate citrate buffer) was added to oxidize the samples for 45 min. Samples were incubated with Ehrlich’s reagent (Sigma, Cat.# D2004) at 65 °C for 45 min. Absorbance was measured at 550 nm using an Infinite M200 spectrophotometer (Tecan), and hydroxyproline concentration was calculated for each sample.

### 2.11 Cytokine measurements

Medium samples were collected at 0.5, 1, 2, and 24 h, and at 14 days (end of conditioning) after POBA or stent implantation, with fully endothelialized TEBVs as controls. Levels of interleukin 6 (IL-6, Cat.# DY206-05), interleukin 8 (IL-8, Cat.# DY208-05), and monocyte chemoattractant factor 1 (MCP-1, Cat.# DY279-05) were assessed using DuoSet ELISA kits (R&D Systems, Minneapolis, USA).

### 2.12 Statistical analysis

Statistical analyses and creation of graphs were performed with GraphPad Prism (GraphPad Software, Version 10.0.2.232). Continuous data are presented as mean ± SD. We used Shapiro-Wilk test to assess normality. Statistical differences were determined with Student’s t test and one-way ANOVA, followed by Tukey multiple comparisons testing for normally distributed variables, and with Mann-Whitney U test and Kruskal-Wallis test followed by Dunn’s multiple comparisons testing for non-normally distributed variables.

## 3 Results

### 3.1 Hemodynamic characterization

Reynold numbers were calculated for all experimental conditions and summarized in Supplementary Table S2. With the exception of TEBVs with incomplete endothelialization and TEBVs without ECs, Re was below 2000, indicating laminar flow.

### 3.2 Impact of endothelial integrity on wall thickening

**FIGURE 3:**
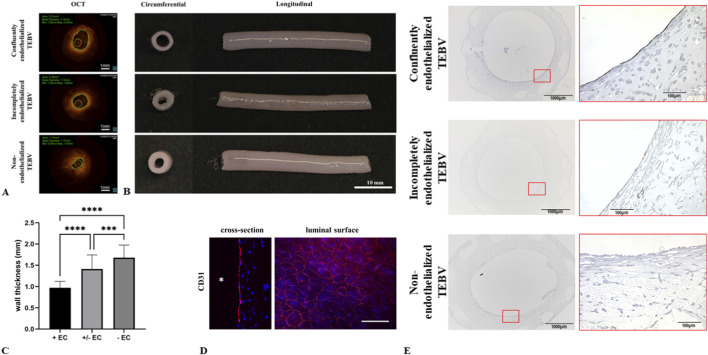
Impact of endothelial integrity on wall thickening **(A,B)** TEBVs with absent or incomplete endothelialization exhibited a reduced lumen diameter compared with TEBVs generated with a confluent EC layer, as verified by OCT **(A)** and macroscopically **(B)**. **(C)** Compared with TEBVs that were confluently seeded with ECs, TEBVs with absent or incomplete endothelial layer exhibited significantly thicker vessel walls (****P < 0.0001; ***P < 0.005). **(D,E)** Immunofluorescence **(D)** and immunohistochemical [**(E)**, upper panel] staining against CD31 confirmed a confluent EC layer on the inner surface of TEBVs seeded with ECs at a concentration of 3 × 10^6^ cells/mL. Endothelialization was incomplete in TEBVs that were seeded with ECs at lower cell concentration [**(E)**, middle panel]. No CD31-positive cells were observed in TEBVs without ECs [**(E)**, lower panel]. All experiments were performed in triplicate (n = 3 per condition). Scale bars: 1000 µm (overview, left), 100 µm (cut-out, right).

To test the effect of endothelial integrity on vascular wall thickening and IH, TEBVs were generated with confluent, semi-confluent, and without an EC layer. After 14 days of dynamic conditioning under arterial flow and pressure conditions, TEBVs underwent OCT-imaging. Introduction of the OCT-catheter and imaging was feasible in all TEBVs. TEBVs with absent or incomplete endothelium showed reduced lumen diameter in OCT compared with confluently endothelialized TEBVs (Figure 3A).

Macroscopically, vessel walls were thicker in TEBVs without ECs (1.68 ± 0.30 mm) than in semi-confluent (1.41 ± 0.33 mm) or fully endothelialized TEBVs (0.97 ± 0.15 mm) (Figures 3B,C). Staining against CD31 confirmed uniform EC coverage in TEBVs seeded with higher EC concentrations, while lower seeding concentration led to incomplete endothelialization (Figures 3D,E).

To further assess the endothelial status, immunofluorescence staining for the endothelial activation markers E-selectin, ICAM-1, and VCAM-1 was performed. Neither marker showed detectable expression in TEBVs with confluent, incomplete, or absent endothelial coverage (data not shown). Staining for eNOS and iNOS was additionally performed to evaluate endothelial function and inflammatory signaling. While eNOS signal was observed, it was not restricted to the luminal surface and was also present in non-endothelialized TEBVs, indicating non-specific background staining. iNOS expression was low across all groups without apparent differences between conditions (Supplementary Figure S1).

### 3.3 Cellular composition and proliferation in TEBVs with intact, semi-confluent, and absent endothelium

**FIGURE 4:**
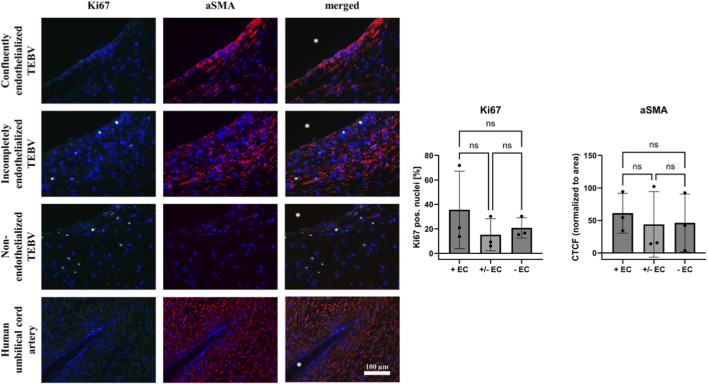
Cellular proliferation and composition of TEBV vessel walls. Representative immunofluorescence images of TEBVs with non-endothelialized, semi-confluent, and fully confluent endothelial layers stained for Ki67 (green) to indicate proliferating cells and αSMA (red) to indicate smooth muscle cell contractile phenotype. Nuclei were counterstained with DAPI (blue). Quantification of %Ki67-positive nuclei relative to total nuclei and CTCF of αSMA normalized to vessel area revealed no statistically significant differences among the groups (p = n.s.), indicating that SMC proliferation and αSMA expression were largely independent of endothelial coverage under the conditions tested. * indicates lumen area.

**FIGURE 5:**
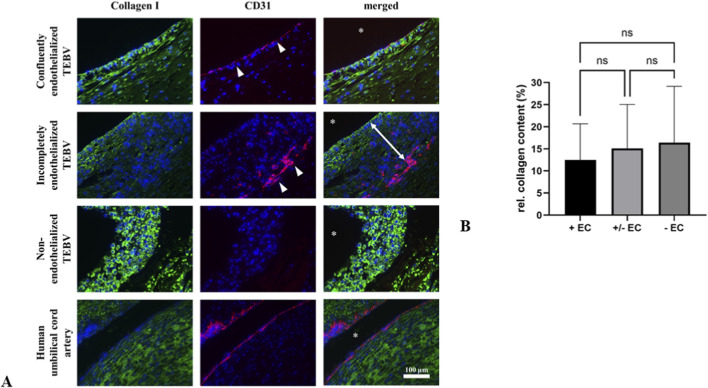
Endothelialization and collagen content in TEBVs **(A)** Immunofluorescence double staining against collagen I and CD31. In TEBVs with a confluent endothelial layer, collagen appeared organized in elongated fibers, and CD31-positive ECs (white arrowheads) formed a continuous, uniform layer on the luminal surface. In TEBVs seeded with ECs at lower cell concentration, CD31-positive cells were found within the TEBV wall, covered by a layer of CD31-negative cells, and collagen was detectable primarily at the luminal side of the vessel wall. Collagen-I staining was most pronounced in non-endothelialized TEBVs, although a clear fibrillar structure was not observed. **(B)** Relative to native arteries (50 μg/mg), collagen content, assessed by hydroxyproline assay, was highest in TEBVs without ECs and lowest in confluently endothelialized TEBVs, with incompletely endothelialized TEBVs in between, however, these differences were not statistically significant (ns = not significant). All experiments were performed in triplicate (n = 3 per condition).

Cellular proliferation and composition of TEBV vessel walls were assessed by immunofluorescence microscopy (Figures 4, 5). To assess cellular proliferation in TEBVs, we performed immunofluorescence staining against Ki67. Quantitative analysis revealed no statistically significant differences in Ki67 expression among TEBVs with non-, semi- or fully confluent endothelium (Ki67% positive nuclei: non-endothelialized 20.76% ± 8.30%; semi-confluent 15.18% ± 13.05%; confluent 35.54% ± 31.71%; p = n.s.) (Figure 4).

Staining against αSMA, a marker indicating SMC contractile properties (Arnoldi et al., 2012; Hinz, 2016), revealed comparable expression levels across all TEBVs, irrespective of endothelial coverage (mean corrected total cell fluorescence [CTCF] ± SD: non-endothelialized 46.16 ± 44.60; semi-confluent 44.00 ± 50.47; confluent 61.07 ± 30.64), indicating that SMC contractile differentiation was maintained in the vessel walls regardless of the presence or absence of an endothelial layer (Figure 4). We further examined SMC activation by quantifying the expression of vimentin, CD68, and CD45 normalized to vessel area (Supplementary Figure S2). Vimentin expression was similar across groups (mean CTCF ± SD: non-endothelialized 92.04 ± 60.26; semi-confluent 102.4 ± 36.21; confluent 83.36 ± 40.15; p = n.s.). CD68 and CD45, both markers of macrophage-like SMCs (Yap et al., 2021), showed generally low expression across all groups, with no statistically significant differences between non-, semi-, or fully endothelialized TEBVs.

### 3.4 Collagen content in TEBVs with intact, semi-confluent, and absent endothelium

Next, to localize and quantify the extent of matrix protein deposition, we performed immunofluorescence staining against collagen I and CD31 (Figure 5A). Interestingly, we found CD31-positive cells within the TEBV wall, covered by a layer of CD31-negative cells in TEBVs seeded with ECs at lower cell concentration. Collagen-I staining appeared more extensive in non-endothelialized TEBVs compared with partially and completely endothelialized samples. In TEBVs with a confluent endothelial layer, collagen appeared organized in elongated fibers. In partially endothelialized TEBVs, such organization was detectable primarily at the luminal side of the vessel wall, whereas non-endothelialized TEBVs exhibited no clear fiber structure.

For quantitative assessment of collagen content in TEBVs, hydroxyproline concentration, a major amino acid constituent of collagen, was measured in samples of TEBVs. In line with our findings from immunofluorescence staining, mean hydroxyproline content was highest in TEBVs without ECs while it was lowest in TEBVs with an intact endothelial layer, albeit without statistical significance (Figure 5B). Relative to the average amount of hydroxyproline found in native arteries (50 μg/mg) (Schmidt et al., 2006), hydroxyproline content was 12.49% ± 8.20% in TEBVs with an intact endothelial layer, 15.12% ± 9.95% in TEBVs with incomplete endothelialization, and 16.41% ± 12.75% in TEBVs without ECs.

### 3.5 Plain-old balloon dilatation and stent implantation of TEBVs

**FIGURE 6:**
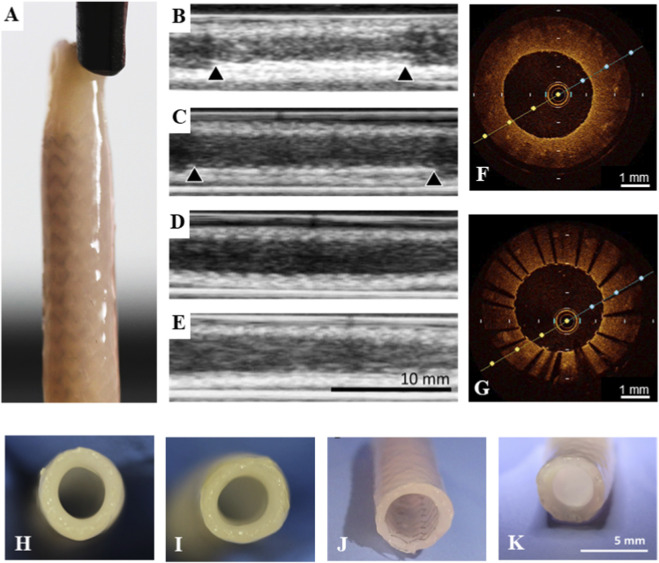
POBA and stent implantation in TEBVs **(A)** TEBV with a commercially available human-sized coronary stent. **(B–E)** Ultrasound monitoring proved useful for non-invasive monitoring after implantation of BMS **(B)** and DES **(C)**, after POBA **(D)**, and in untreated control TEBVs **(E)**. Boundaries of the stents (black arrowheads) as well as the TEBV vessel walls were clearly visible. **(F,G)** OCT-imaging was performed 14 days after POBA and stent implantation. **(H–K)** Macroscopic views of control **(H)**, POBA-treated **(I)**, BMS-treated **(J)**, and DES-treated **(K)** TEBVs.

To assess whether TEBVs could serve as *in vitro* models for the evaluation of coronary devices, we performed POBA and stent implantation (Figure 6A) in TEBVs at 7 days of conditioning. Non-treated TEBVs seeded with a confluent layer of ECs served as controls (all n = 3). All procedures were carried out successfully, with adequate trackability of balloons and balloon-mounted coronary stents over a guidewire. No TEBVs were obviously damaged during the procedure. Over the course of the conditioning period of 14 days after the procedures (*i.e*., 21 days in total), no infection occurred, and all bioreactors remained sterile. Ultrasound monitoring was carried out in all TEBVs and proved useful for non-invasive monitoring as boundaries of both the stents and of TEBV walls were clearly definable (Figures 6B–E). TEBVs remained patent throughout the observation period, and stents maintained their original position. In addition, at 14 days of conditioning after POBA and stent implantation, all TEBVs underwent OCT-imaging (Figures 6F,G). Incipient cellular growth was visible above the stent struts as small reflection points. Following OCT-imaging, TEBVs were removed from the bioreactor for macroscopic inspection (Figures 6H–K).

### 3.6 Histology of TEBVs after POBA and stent implantation

**FIGURE 7:**
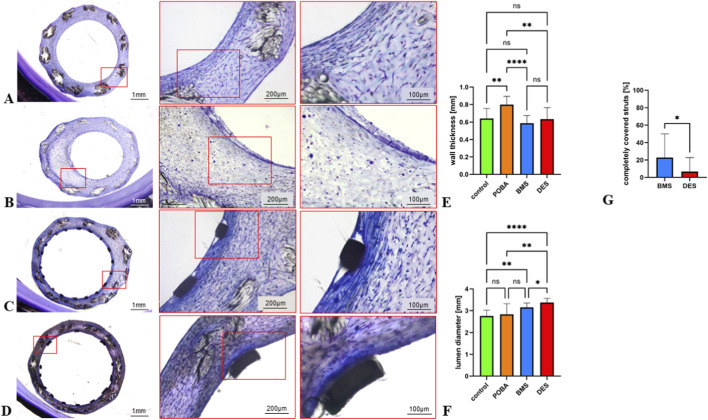
Histology of stented and POBA-treated TEBVs compared with untreated control TEBVs **(A–D)** Untreated control **(A)**, POBA- **(B)**, BMS- **(C)**, and DES- **(D)** treated TEBVs were embedded in plastic and processed for histology (toluidine blue/fuchsine staining). **(E)** Vessel wall thickness was higher in POBA-treated TEBVs compared with untreated TEBV controls and stented TEBVs. **(F)** Lumen diameter was greater in stented compared with POBA-treated and untreated control TEBVs. DES-treated TEBVs exhibited the largest lumen diameter. No significant difference in lumen diameter was observed between BMS- and POBA-treated TEBVs, or between POBA-treated TEBVs and untreated controls. **(G)** 14 days after stent implantation, 22.9% of struts were completely covered with neointima in BMS-treated TEBVs, while it were only 6.8% of struts in DES-treated TEBVs. All experiments were performed in triplicate (n = 3 per condition). ****P < 0.0001; ***P < 0.005, **P < 0.01, *P < 0.05.

TEBVs were embedded in PMMA and processed for histology (Figures 7A–D). Vessel wall thickness was highest in POBA-treated TEBVs (0.8 mm ± 0.1 mm) compared with untreated TEBV controls (0.64 mm ± 0.11 mm) and compared with BMS- (0.59 mm ± 0.09 mm) and DES- (0.63 mm ± 0.13 mm) treated TEBVs (p < 0.001, Figure 7E). There were no significant differences in vessel wall thickness among other comparison groups. In particular, wall thickness was comparable between BMS- and DES-treated TEBVs.

Lumen diameter was greater in stented TEBVs compared with POBA-treated (2.83 mm ± 0.49 mm) and untreated control TEBVs (2.75 mm ± 0.27 mm), with DES-treated TEBVs exhibiting the largest lumen (3.38 mm ± 0.19 mm), which was also significantly larger compared with BMS-treated TEBVs (3.15 mm ± 0.21mm, Figure 7F). No significant difference was observed between BMS- and POBA-treated TEBVs, or between POBA-treated and untreated controls.

We found incipient neointima formation around the stent struts within TEBVs treated with BMS (Figure 7C). In contrast, there was only marginal neointimal growth within TEBVs treated with DES (Figure 7D). In cross-sections of BMS-treated TEBVs, 22.9% of struts were completely covered with neointima, while it was only 6.8% of struts in DES-treated TEBVs (Figure 7G).

### 3.7 Inflammatory cytokine levels following POBA and stent implantation

**FIGURE 8:**
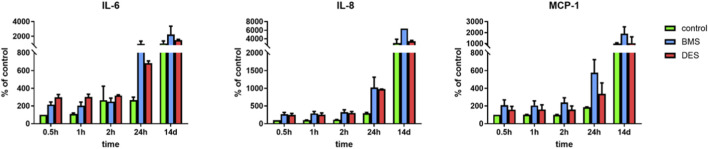
Cytokine levels in culture medium samples from stented vs. untreated control TEBVs. IL-6, IL-8, and MCP-1 levels increased over time in culture medium from stented and control TEBVs, with highest levels in BMS-treated TEBVs, however, no statistically significant differences were observed among BMS, DES, and control TEBVs at each time point. All experiments were performed in triplicate (n = 3 per condition).

Over the course of the first 24h of the conditioning period after stent implantation, we observed a steady increase of levels of IL-6, IL-8, and MCP-1 in culture medium samples from stented and untreated control TEBVs (Figure 8). Cytokine levels at 14 days were significantly higher compared to baseline levels in all samples. The highest levels of IL-6, IL-8, and MCP-1 in culture medium were obtained from bioreactors with BMS-treated TEBVs. There were no significant differences in IL-6-, IL-8-, and MCP-1-levels between DES-treated TEBVs and untreated controls at 14 days.

## 4 Discussion

Here, we present TEBVs as an *in vitro* model for IH and restenosis following POBA and stent implantation. Owing to its tubular shape and its dimensions similar to a human coronary artery, our model not only enables the investigation of EC-SMC crosstalk under shear stress and under different culture conditions, but also allows for POBA and for the implantation of human-sized coronary stents.

EC-SMC crosstalk is crucial to maintain vascular homeostasis. An intact EC layer is essential for the integrity of the vessel wall and contributes to the quiescent state of the underlying SMCs (Lilly, 2014; Fillinger et al., 1997; Powell et al., 1998; Korff et al., 2001). ECs also inhibit SMC type I collagen synthesis when co-cultured as a bilayer (Powell et al., 1998). Vice versa, perturbations in the EC-SMC crosstalk can elicit endothelial dysfunction or phenotypic switching of SMCs from a contractile and quiescent towards a synthetically active, migratory and proliferative phenotype (Lilly, 2014).

Three-dimensional co-culture models have the potential to replicate not only the anatomical macrostructure but also the microstructure at tissue-and at cell-level, including ECM deposition and arrangement. Therefore, three-dimensional models such as EC-SMC spheroids with direct cultures of ECs and SMCs (Mallone et al., 2018), vascular organoids created from induced pluripotent stems cells (Wimmer et al., 2019a), and cultures of ECs on ECM-like gels containing SMCs (Dorweiler et al., 2006) have increased physiological relevance and may bridge the gap between traditional 2D cell culture and *in vivo* animal models (Fitzgerald et al., 2015). However, these models are not able to recapitulate the hemodynamic environment of blood vessels. Under static conditions, ECs induce SMC migration in a co-culture system by increased endothelial expression of platelet-derived growth factor (Hsieh et al., 1992) Shear stress, however, induces the release of nitric oxide in ECs, which inhibits SMC migration (Garanich et al., 2005; Rojas-González et al., 2023). Human blood vessels are continuously exposed to mechanical stimuli such as blood pressure and shear stress, and sustained exposure of the human vascular wall to non-physiological flow and pressure conditions may elicit changes in the proliferative and migratory status of cells within the vessel wall, eventually leading to vascular remodeling (Méndez-Barbero et al., 2021). Therefore, we generated TEBVs as dynamic 3D models which can be exposed to mechanical stimuli. TEBVs in our model were conditioned in a bioreactor ensuring a continuous exposure to physiological flow and pressure conditions. In our model, for most conditions Re was below 2000, which corresponds to a laminar flow.

### 4.1 TEBVs as *in vitro* models for the research on atherosclerosis

The introduction of triple-layered TEBVs in 1998 (L'Heureux et al., 1998) as well as pioneering work by Niklason et al. (1999) laid the foundation for tremendous research activities to further refine TEBV development with both autologous (Kelm et al., 2012) and allogenic cells (Wystrychowski et al., 2014). Originally intended for the use as vascular grafts for coronary artery bypass surgery, for the treatment of peripheral artery disease, or for hemodialysis access (Wystrychowski et al., 2014; Peck et al., 2012), TEBVs have also been evaluated as models for *in vitro* atherosclerosis research (Robert et al., 2013), and as testing platforms for vascular interventions such as POBA and stent implantation (Cardinal et al., 2006; Cardinal and Williams, 2009).

Many aspects of EC-SMC interactions have been studied previously in TEBVs. Early experiments by Niklason et al. demonstrated decreased SMC proliferation in endothelialized vessels as compared with vessels without EC (Niklason et al., 1999). Another study demonstrated that long-term (15 days) co-cultures of ECs and SMCs in porous tubular PGA scaffolds downregulated collagen and proteoglycan deposition, with SMCs exhibiting a more contractile phenotype compared to 2-day co-culture constructs (Williams and Wick, 2005). Moreover, micropatterned co-culture of vascular ECs and SMCs on bilayered electrospun fibrous mats produced high alignment of extracellular matrix in circumferential direction (Liu et al., 2015).

More than 20 years ago, TEBVs with a contractile vascular media were developed from sheets of human SMCs wrapped around a styrene tubular support and displayed contraction and relaxation in response to vasoconstrictor and vasodilator agents (L'Heureux et al., 2001). Later on, Schmidt et al. presented TEBVs generated from myofibroblasts seeded on biodegradable polyglycolic-acid scaffolds and lined with ECs that were exposed to flow conditions (Schmidt et al., 2006). A decade ago, the group suggested their constructs as a model to study essential pathogenic phenomena of atherosclerosis *in vitro* (Robert et al., 2013). They were able to demonstrate enhanced adhesion and transmigration of monocytes injected into the medium upon endothelial stimulation with either tumor necrosis factor α or low-density lipoprotein cholesterol (LDL-C). This model represented a significant step towards a relevant *in vitro* platform for the systematic assessment of atherosclerotic pathways. However, with hydroxyproline content at only 1% of that in native arteries (Schmidt et al., 2006), the model exhibits lower collagen levels and only partially reflects the composition of human coronary arteries. Furthermore, the model was equipped with a tubular backbone scaffold of PGA for mechanical support, which introduces mechanical stiffness of the vessel wall and reduces vascular compliance.

To overcome these limitations and improve biomimicry, we developed coronary-scale TEBV composed of a fibrin gel matrix supported by a macroporous textile mesh. Fibrin gel not only enables a uniform cell distribution but has also been shown to promote the synthesis of matrix proteins by seeded cells, including elastin and collagen (Grassl et al., 2002; Long and Tranquillo, 2003; Koch et al., 2010). As we have demonstrated before, the mesh in our TEBVs provided adequate mechanical support without limiting the resilience and compliance of the vessel wall (Tschoeke et al., 2008; Fernández-Colino et al., 2019), thereby enabling a vessel wall that more closely reflects the native human coronary artery in structure and behavior.

In addition, our study introduces a systematic comparison of three distinct levels of endothelial coverage–fully confluent, semi-confluent, and absent – within flow-conditioned TEBVs composed entirely of human cells. This allows direct assessment of the impact of endothelial integrity on vessel wall remodeling. Compared with other *in vitro* vascular models, our TEBV system combines several key features that enhance its physiological relevance. While other TEBVs often rely on rigid scaffold materials (Schmidt et al., 2006) or lack a fully developed medial layer (Cardinal et al., 2006), microfluidic platforms are typically limited to small vessel diameters and simplified wall architecture (Zheng et al., 2012; Choi and Seo, 2019), and organoid-based systems lack defined geometry and hemodynamic conditioning (Wimmer et al., 2019a; Wimmer et al., 2019b; Kahn-Krell et al., 2022; Trillhaase et al., 2021). In contrast, our model integrates a fully human, three-dimensional vessel wall with embedded SMCs, human coronary artery dimensions, and physiological flow conditions, while enabling the use of clinically relevant interventional devices such as balloons and stents (Table 1).

**TABLE 1:** Comparison of *in vitro* vascular models.

​	Conventional TEBVs	Microfluidic/organ-on-chip models	Organoid-based systems	This study (TEBV)
Vessel wall architecture	3D, scaffold-dependent	Simplified, often 2D/monolayer	Heterogeneous, mostly 3D	3D, multilayered (ECs + SMCs)
Cellular composition	Human or animal cells	Mostly human ECs ± supporting cells	Human (induced) pluripotent stem cells	Human ECs + SMCs
Mechanical properties	Scaffold-dependent	Limited physiological compliance	Not well defined	Compliant, fibrin-based matrix with textile support
Hemodynamic conditions	Variable	Precisely controlled flow	Not defined; no perfusion *in vitro*	Physiological pulsatile flow
Vessel size	Mm-scale	µm-scale	µm-scale	Mm-scale
Suitability for stent testing	Variable	Not feasible	Not feasible	Feasible
Application focus	Grafts, basic biological research	Mechanistic studies	Developmental biology	IH, device testing

While replication of SMCs has been shown to be inhibited by confluent ECs in co-culture experiments (Dodge et al., 1993), quantitative analysis of Ki67 in our TEBVs revealed no statistically significant differences in proliferative activity across varying levels of endothelial coverage. This suggests that proliferation dynamics are more complex than a simple inverse relationship with endothelial integrity, and that additional mechanisms beyond SMC proliferation likely contribute to vessel wall remodeling. Similarly, αSMA expression, a marker of SMC contractile phenotype (Arnoldi et al., 2012; Hinz, 2016), and vimentin, an intermediate filament protein indicative of SMC synthetic activity (Beamish et al., 2010), showed comparable expression levels across all groups, suggesting that baseline SMC differentiation is maintained in TEBVs, irrespective of the endothelial state. CD68 and CD45, both indicative of macrophage-like or activated SMCs (Yap et al., 2021), were detectable in all TEBVs but did not differ significantly across groups, suggesting that endothelial coverage in this model does not strongly modulate SMC differentiation toward a macrophage-like phenotype.

While IH *in vivo* is often associated with endothelial activation and subsequent SMC activation, our TEBV model did not show evidence of classical endothelial activation or robust SMC activation. I contrast, collagen deposition was increased in TEBVs without ECs and in TEBVs with incomplete endothelialization, and these TEBVs had thicker vascular walls and reduced lumen diameters, reflecting extracellular matrix remodeling associated with disrupted endothelial coverage.

Taken together, these findings indicate that endothelial integrity in TEBVs influences vessel wall remodeling primarily through effects on extracellular matrix deposition, rather than through endothelial activation or changes in SMC proliferation, contractile differentiation, or macrophage-like activation. This highlights the complex interplay between endothelial coverage, matrix remodeling, and SMC phenotypic plasticity, recapitulating key features of early intimal hyperplasia in a human-relevant *in vitro* system.

In TEBVs with incomplete endothelialization, CD31-positive ECs did not mark the inner lining but were found within the vascular wall, covered by newly-formed tissue consisting of collagen fibers and of cells that stained negative for αSMA. This pattern resembles the characteristic image of IH, where a gradual diminution of the vessel lumen occurs due to migration and proliferation of SMCs into the vascular intima (Mills et al., 2012). IH is the most frequent cause for vessel failure subsequent to aortocoronary vein grafting (Wilbring et al., 2011) and vascular interventions such as POBA and stent implantation (Curcio et al., 2011), and the principal stimulus for IH appears to be endothelial injury. However, the exact mechanisms by which IH occurs are incompletely understood. Taken together, the combination of a biomimetic human vessel wall, graded endothelial integrity, and relevant hemodynamic conditioning establishes our TEBV as a promising *in-vitro* system for mechanistic studies of atherosclerosis and IH that more closely reflects early human coronary pathology.

### 4.2 TEBVs as a platform for intravascular device testing

O’Halloran-Cardinal et al. were the first to demonstrate that stent implantation as well as OCT imaging were feasible in TEBVs composed of an ePTFE scaffold with an intimal lining of human microvessel ECs (Cardinal et al., 2006). Some years later, the group was able to show accelerated regeneration of ECs over protein-modified stent strut surfaces with significantly increased tissue thickness compared to BMS (Cardinal and Williams, 2009), supporting the use of TEBVs for evaluation of modified stent surfaces. Their model, however, lacked a medial layer, although some interspersed SMCs were noted below the endothelium, possibly because cells were isolated from liposuction fat, resulting in a mixed population of cells with mesenchymal and endothelial origin. Moreover, the compliance of the TEBV wall was limited due to the polymer scaffold employed to support the ECs. Therefore, this system has limited ability to fully mimic features of human blood vessels.

O’Cearbhaill et al. developed a more compliant TEBV with a backbone of silicone seeded with ECs which allowed for the incorporation of pressure, tensile hoop strain, and shear stress into the model (O'Cearbhaill et al., 2008). The group was able to demonstrate enhanced expression of genes indicating an inflammatory response in ECs at 24h after stent implantation, including E-Selectin, ICAM-1, and VCAM-1, along with an increased expression of the pro-apoptotic protein Bax and a decrease in the anti-apoptotic protein Bcl-2 and in Ki67. Although this model had improved ability to mimic hemodynamic forces, the absence of SMCs and ECM proteins within the vascular wall was a major limitation. Furthermore, the group used human umbilical vein ECs (HUVECs) instead of arterial ECs. However, ECs from different vascular beds exhibit distinct functional and phenotypic properties. Arterial ECs are exposed *in vivo* to higher shear stress and pulsatile pressure, and they display differential gene expression, inflammatory responses, and susceptibility to atherosclerotic stimuli compared with venous ECs (Ohura et al., 2003; Cox and Ng, 2025). Using arterial-derived EC therefore allows our TEBV model to more accurately mimic the mechanical and biological environment of coronary arteries, providing more physiologically relevant insights into EC-SMC crosstalk, vascular remodeling, and responses to interventions such as POBA or stent implantation.

In summary, while stent deployment has been technically demonstrated in TEBVs before, our study provides the first human-cell, flow-conditioned, coronary-scale TEBV with a medial SMC layer, capable of modeling early neointimal responses to clinically relevant stent types and POBA, and amenable to longitudinal imaging via OCT and ultrasound. This represents a substantial advance in TEBV design for *in vitro* evaluation of coronary devices.

### 4.3 Inflammatory responses and endothelial healing following POBA and stent implantation in TEBVs

In line with findings from O'Cearbhaill et al. (2008), we observed an inflammatory response in our model, as indicated by increasing levels of proinflammatory cytokines within the culture medium over the course of 12 days after stent implantation. Highest levels of IL-6, IL-8, and MCP-1 were observed in culture medium samples from BMS-treated TEBVs, albeit without statistical significance.

Mechanical injury, such as POBA and stent implantation, elicits an inflammatory response from the vessel wall, including the recruitment of monocytes and other leukocytes to areas of vascular injury, which is mediated by chemoattractant cytokines and interleukins (Welt and Rogers, 2002). Cipollone et al. have demonstrated elevated levels of MCP-1 in patients with restenosis after POBA (Cipollone et al., 2001), and increased levels of IL-6 and IL-8 have been found after implantation of BMS and DES (Li et al., 2020). However, comparisons of cytokine levels following BMS- or DES-implantation in humans yielded conflicting results. The anti-proliferative drug-coating of DES (*i.e.*, everolimus in our study), possesses anti-inflammatory properties, as evidenced by their current use as immunomodulatory agents in the prevention and treatment of transplant rejection (Tedesco-Silva et al., 2019). Indeed, in a porcine model of stent implantation, Suzuki et al. found reduced expression of MCP-1 and IL-6 within the vessel wall after DES-implantation compared to the expression after BMS-implantation (Suzuki et al., 2001), and everolimus-eluting stents have been suggested to stabilize plaque inflammation *in vivo* (Calfon Press et al., 2017). Other studies reported a local inflammatory response following first-generation DES-implantation (Joner et al., 2006), and – compared with BMS-treated patients – systemic inflammatory responses were accentuated in patients undergoing DES-implantation for acute coronary syndrome (Liu et al., 2014).

We also observed a progressive increase in inflammatory cytokines in control TEBVs. This likely reflects baseline cellular responses to culture conditions and tissue remodeling rather than injury-induced inflammation. Several mechanisms may contribute: endothelial activation by culture-related stress (e.g., non-physiological shear stress, nutrient fluctuations, or contact with biomaterials); dynamic remodeling and maturation processes including proliferation of ECs and SMCs, ECM synthesis, and matrix turnover, which can trigger intrinsic cytokine signaling; and biomaterial-induced low-grade inflammation mediated by mechanosensitive pathways. Additionally, closed-loop culture may lead to cytokine accumulation over time in the absence of systemic clearance. Minor contributions from apoptotic cells or endothelial-to-mesenchymal transition may further enhance IL-6 and IL-8 secretion.

Although cytokine levels in the controls were lower than in mechanically injured TEBVs, these findings indicate that the constructs are metabolically active and responsive to environmental cues, supporting the validity of our model. Nevertheless, while endothelial cells contribute to inflammatory reactions following vascular injury, the bulk of proinflammatory cytokines in humans is released by leukocytes. Thus, our model does not fully account for inflammation in its current form. Further studies including circulating lymphocytes are warranted to investigate inflammatory responses in TEBVs upon POBA and stent implantation.

In line with findings from clinical and histopathological studies (Joner et al., 2006; Cornelissen et al., 2021), we observed delayed endothelial healing in TEBVs after DES-implantation, whereas more than 20% of stent struts were covered by neointima in BMS-treated TEBVs 12 days after the procedure. The antiproliferative drug-coating, intended to reduce neointimal formation by impeding SMC proliferation and migration, also impairs the normal healing processes of the injured arterial wall (Marx et al., 1995).

Nevertheless, DES demonstrate a significant reduction in restenosis compared with BMS, and over the last decades, design and biocompatibility of DES have been evolving rapidly (Torii et al., 2020). Latest-generation DES are characterized by improved stent architecture with thinner strut backbones, increased radial strength and improved deliverability (Mori et al., 2021). They also have improved durable and biodegradable polymers – and in some cases no polymer – as well as new antiproliferative drug types and dosing. However, the issue of late in-stent restenosis, including neoatherosclerosis after DES implantation, is still to overcome (Madhavan et al., 2020). Further research should focus on strategies to minimize vascular injury and to improve biocompatibility of DES by material innovation, titration of drug-release and polymer degradation profile, and minimized strut thickness. Therefore, DES will continue to evolve, and TEBVs might potentially allow for the initial *in vitro* testing of novel stent devices.

### 4.4 Limitations

Our model recapitulates key features of intimal hyperplasia and provides a promising platform for studying human vascular biology and atherosclerotic mechanisms *in vitro*. However, several limitations should be acknowledged. First, atherosclerosis is a multifactorial disease, and while our model reproduces important aspects of early vascular remodeling other critical components remain unaddressed. TEBVs were perfused with cell culture medium only and were not exposed to the cellular and molecular constituents of human blood. Consequently, important hemodynamic and biochemical factors, including turbulent flow, shear stress alterations, lipid accumulation, leukocyte adhesion and transmigration, were not modeled. Notably, circulating blood cells, which are thought to contribute to in-stent restenosis (Bayes-Genis et al., 2002), were absent. Due to the pulsatile nature of the flow, we acknowledge the possibility of local variations in shear stress and consider this, as well as the lack of compliance measurements as a limitation of our study. The here reported Re indicated laminar flow for most conditions. The Re for TEBVs with a semi-confluent EC layer and those with no EC layer, fall within the transitional regime (2000 < Re < 4000), where flow is not fully turbulent but might present intermittent instabilities. A similar situation can occur *in vivo*, when pathological changes in blood vessels (e.g., wall thickening) leading to luminal narrowing can result in local and intermittent fluctuations of shear stress and flow velocity rather than a fully developed turbulent flow (Chiu and Chien, 2011). Furthermore, although quantitative analyses of SMC proliferation (Ki67), contractile differentiation (αSMA), activation and migration (vimentin), and macrophage-like phenotype markers (CD68, CD45) were performed, no statistically significant differences were observed across TEBVs with non-, semi-, or fully confluent endothelium. These findings indicate that endothelial integrity in this system does not strongly modulate baseline SMC proliferation, contractile differentiation, or macrophage-like activation, and that vessel wall remodeling likely involves additional factors beyond simple endothelial coverage. Due to the use of primary human cells, the number of biological replicates in this study was limited. While technical replicates were included and culture conditions were carefully standardized to maximize reproducibility, this sample size constrains statistical power. Future studies with larger numbers of donors will be important to confirm and extend these findings. Another limitation is the maximum conditioning period of 21 days, which does not reflect the long-term development of intimal hyperplasia, in-stent restenosis, or atherosclerosis that typically develop over years.

Despite these limitations, the combination of a biomimetic human vessel wall, graded endothelial coverage, and physiologically relevant hemodynamic conditioning provides a robust platform for mechanistic studies of early vascular remodeling and preclinical testing of cardiovascular devices.

## 5 Conclusion

Our three-dimensional TEBV model composed of SMCs and ECs recapitulates fundamental principles of vascular biology. While quantitative analyses of SMC proliferation, contractile differentiation, and macrophage-like activation did not differ significantly across TEBVs with varying endothelial coverage, vessel morphology and extracellular matrix deposition (particularly collagen accumulation) indicate that endothelial integrity influences vessel wall remodeling.

Moreover, POBA as well as the implantation of human-sized coronary stents was feasible with TEBVs in our model, as they are similar in size and wall thickness to human coronary arteries.

Taken together, these findings highlight TEBVs have the potential to serve as a platform for mechanistic studies of intimal hyperplasia, and may be used for high-throughput preclinical testing of cardiovascular devices.

## Data Availability

The raw data supporting the conclusions of this article will be made available by the authors, without undue reservation.
