# Supplementary figures and images for: Evaluation of tissue-engineered blood vessels as three-dimensional in vitro testing system in cardiovascular research
Source: Front Bioeng Biotechnol. 2026 May 18;14:1729469. doi: 10.3389/fbioe.2026.1729469 (PMC13223119; doi:10.3389/fbioe.2026.1729469)

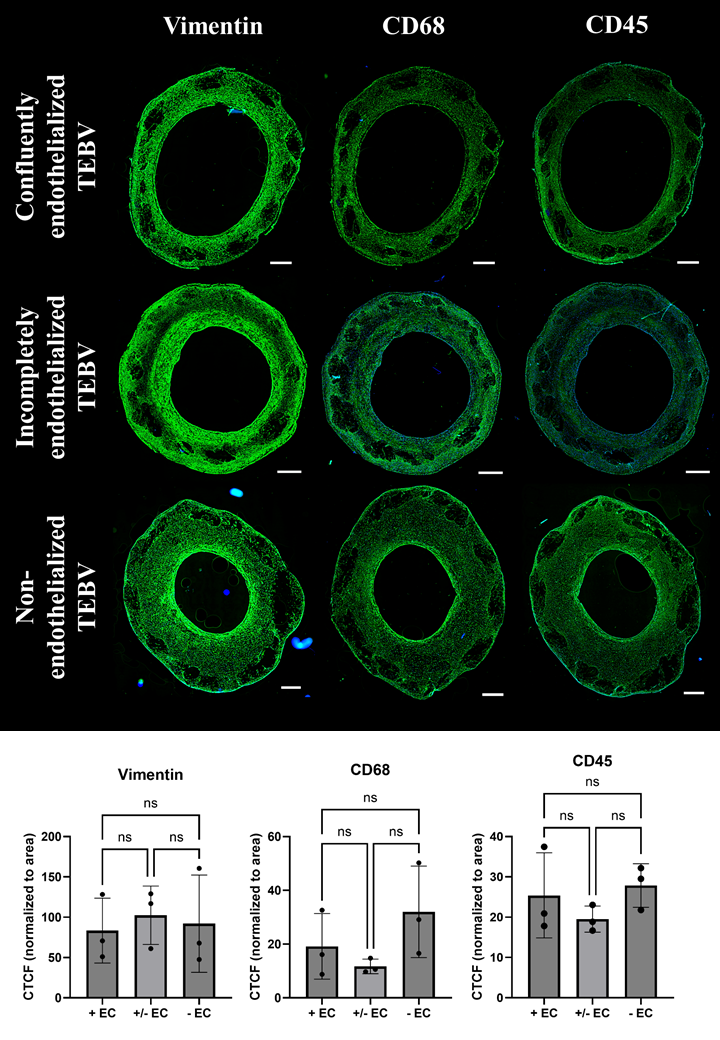

Supplement: Supplementary file 1 [file Image2.tif]

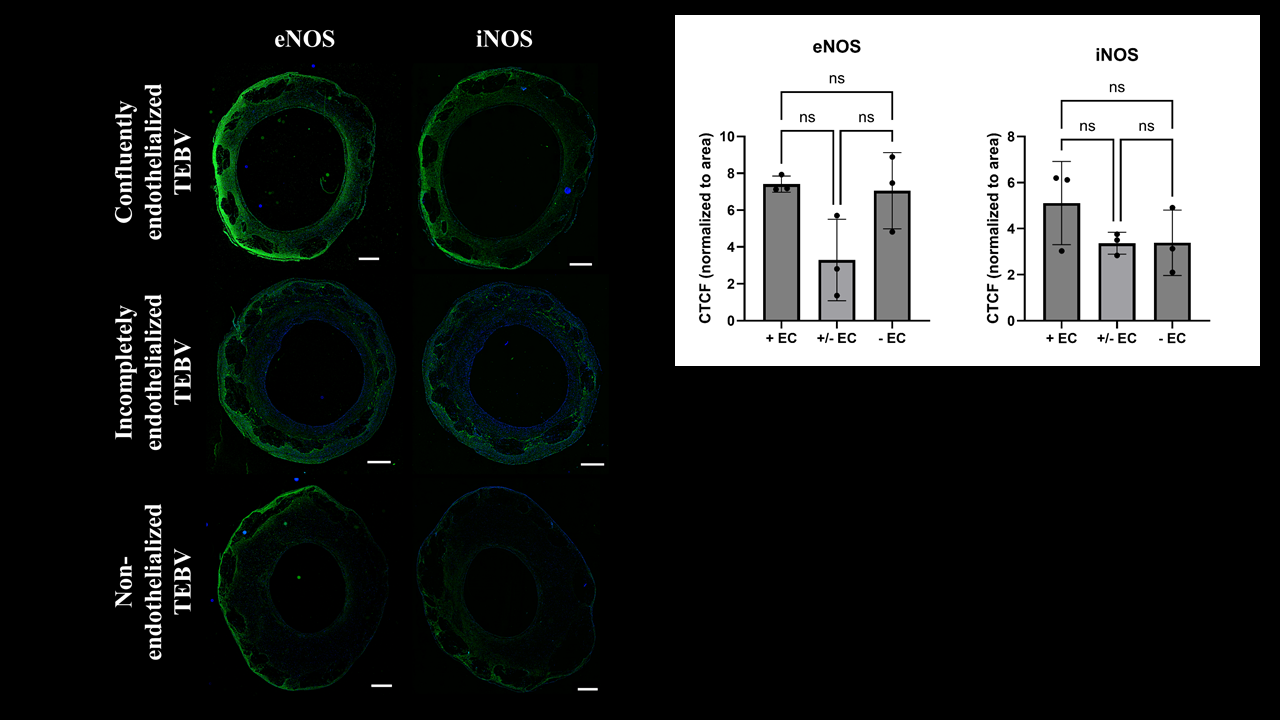

Supplement: Supplementary file 2 [file Image1.tif]
