# Supplementary material for: Evaluation of tissue-engineered blood vessels as three-dimensional in vitro testing system in cardiovascular research
Source: Front Bioeng Biotechnol. 2026 May 18;14:1729469. doi: 10.3389/fbioe.2026.1729469 (PMC13223119; doi:10.3389/fbioe.2026.1729469)
Supplement: Supplementary file 3 [file DataSheet1.docx]

Supplemental Material

# Supplemental Figures

## Supplemental Figure 1 – Staining against eNOS and iNOS.

Staining for eNOS and iNOS was additionally performed to evaluate endothelial function and inflammatory signaling. eNOS signal was observed, however, it was not restricted to the luminal surface and was also present in non-endothelialized TEBVs, indicating non-specific background staining. iNOS expression was low across all groups without apparent differences between conditions

## Supplemental Figure 2 – Immunofluorescence staining for markers of SMC activation in TEBVs with varying endothelial coverage.

**A)** Representative immunofluorescence images of TEBVs with confluent, semi-confluent, and absent endothelial layers stained for vimentin, CD68, and CD45. Vimentin was used as an intermediate filament marker associated with SMC phenotypic modulation, while CD68 and CD45 indicate macrophage-like phenotypes. Nuclei were counterstained with DAPI. Scale bars: 500 µm.

**B)** Signal intensities (CTCF) normalized to vessel area showed no statistically significant differences among groups for any of the markers (p=n.s.), indicating comparable levels of SMC activation and macrophage-like features irrespective of endothelial coverage.

# Supplemental Tables

## Supplemental Table 1: Balloon Catheter and Stent Specifications

| **Device** | **Brand** | **Company** | **Diameter (mm)** | **Strut Thickness (µm)** | **Nominal Pressure (atm)** |
| --- | --- | --- | --- | --- | --- |
| Coronary Dilatation Catheter | TREK™ | Abbott, Chicago, IL | 3.5 | N/A | 8 |
| Bare Metal Stent | Coroflex® Blue Neo | B. Braun | 3.5 | 60 | 10 |
| Drug-Eluting Stent | Promus Element™ Plus | Boston Scientific | 3.5 | 81 | 11 |

## Supplemental Table 2: Reynold numbers for the different experimental conditions

| **Experimental Conditions** | | **Mean luminal diameter (mm)** | **Re** |
| --- | --- | --- | --- |
| **Degrees of Endothelialization** | Confluent EC layer | 2.80 | 1895 |
|  | Semiconfluent EC layer | 2.31 | 2296 |
|  | No EC layer | 1.97 | 2693 |
| **Stent Implantation** | Untreated control | 2.75 | 1929 |
|  | POBA | 2.83 | 1875 |
|  | BMS | 3.15 | 1684 |
|  | DES | 3.38 | 1570 |

## 
